# Effectiveness of Surgical Intervention Compared to Antibiotic Therapy in Managing Localized Diverticular Perforation in Adults: A Systematic Review and Meta-Analysis

**DOI:** 10.7759/cureus.96879

**Published:** 2025-11-15

**Authors:** Mohammad Shiraz, Muneeba Bint E Saeed, Shabih Zahra

**Affiliations:** 1 General Surgery, Peterborough City Hospital, North West Anglia NHS Foundation Trust, Peterborough, GBR; 2 Renal Transplant, Royal Liverpool University Hospital, Liverpool, GBR; 3 Cardiology, Ysbyty Gwynedd Hospital, Bangor, GBR

**Keywords:** antibiotic therapy, diverticular perforation, localized diverticulitis, surgical intervention, systematic review

## Abstract

The optimal management of localized diverticular perforation remains uncertain, with ongoing debate between surgical intervention and conservative antibiotic therapy. While randomized trials have addressed uncomplicated diverticulitis, evidence specific to perforated disease is limited and heterogeneous. This study aimed to evaluate the effectiveness of surgical intervention compared to antibiotic therapy in managing localized diverticular perforation in adults, focusing on key clinical outcomes including adverse events, stoma creation, mortality, and intra-abdominal abscess formation. We conducted a systematic review and meta-analysis of studies comparing surgical versus antibiotic therapy in adults with localized diverticular perforation. Major outcomes included composite adverse events, stoma creation, all-cause mortality, and intra-abdominal abscess. Pooled odds ratios (ORs) with 95% confidence intervals (CIs) were calculated using random-effects models. Heterogeneity was assessed using the I² statistic. Four comparative studies (n = 12,922 patients) were included. For composite adverse outcomes, no significant pooled difference was observed (Pooled OR 3.79, 95% CI 0.36-39.56, p = 0.266; I² = 97.4%), though individual studies showed conflicting results. Surgical intervention was associated with a significantly higher risk of stoma creation compared to antibiotics (Pooled OR 16.16, 95% CI 4.11-63.63, p < 0.001; I² = 43.3%). Mortality did not differ significantly between groups (Pooled OR 3.68, 95% CI 0.28-47.99, p = 0.320; I² = 88.8%). Intra-abdominal abscess rates were also comparable (Pooled OR 3.01, 95% CI 0.53-17.07, p = 0.212; I² = 87.8%). Surgical management of localized diverticular perforation provides definitive treatment but significantly increases the risk of stoma creation. Antibiotic therapy may be effective in carefully selected patients, particularly when initiated promptly, although study findings remain inconsistent. Given the high heterogeneity across available studies, further randomized trials are needed to establish evidence-based guidance for treatment selection in this patient population.

## Introduction and background

Diverticulosis of the colon increases with age, affecting nearly half of individuals over the age of 60 years, while it remains relatively uncommon before the age of 40 [1]. Although the majority of patients remain asymptomatic, approximately one-fifth develop symptoms requiring medical attention during their lifetime [1]. With advancing age and the global increase in life expectancy, the incidence of acute diverticulitis continues to rise, making it a significant burden in clinical practice, particularly in older adults [1,2]. The condition predominantly involves the sigmoid colon, though it may also present in younger patients, often with a more severe clinical course [1,2]. Currently, acute diverticulitis is recognized as one of the most common causes of abdominal emergencies in the elderly population [2].

Over the past decade, both the understanding of sepsis and the management strategies for diverticulitis have undergone considerable change. The Hinchey classification and its subsequent modifications remain widely used in clinical practice to grade the severity of the disease [3-5]. Localized perforation, corresponding to Hinchey stage Ia/Ib, is of particular clinical interest, as it represents an intermediate stage where management strategies remain debated. Immunosuppressed individuals, including those on chronic corticosteroids or with comorbid conditions such as neoplasia, chronic renal or hepatic failure, or HIV/AIDS, are at increased risk of perforation. In such patients, the disease often presents with subtle symptoms, leading to delayed diagnosis and worse outcomes, with morbidity and mortality rates reported as high as 19%-25%, compared to 4%-6% in the general population [5].

Perforation occurs in up to 10% of patients with acute diverticulitis [3]. Several risk factors, including elevated C-reactive protein, leukocytosis, diffuse abdominal pain, delayed diagnosis, and comorbidities, have been associated with higher rates of complicated disease [3,6]. Differences in anatomical distribution have also been reported, with right-sided perforation more common in younger patients and left-sided disease predominating in older adults [6]. Obesity, particularly visceral adiposity, further increases the likelihood of perforation and other complications, although its impact on postoperative outcomes remains debated [1,3,7].

Given the clinical and economic burden of diverticular disease, estimated to exceed two billion dollars annually in the United States alone [5], optimizing management strategies is of major importance. Traditional treatment for localized perforation has included urgent surgical intervention, such as resection or lavage, aimed at preventing progression to generalized peritonitis. However, emerging evidence suggests that carefully selected patients may be managed conservatively with antibiotic therapy, potentially avoiding the risks of surgery without compromising outcomes. The European Association for Endoscopic Surgery (EAES), the Society of American Gastrointestinal and Endoscopic Surgeons (SAGES), and the World Society of Emergency Surgery (WSES) have issued guidelines in recent years that acknowledge both surgical and conservative strategies as valid options in certain cases, reflecting ongoing debate in the field [8,9].

Despite these evolving guidelines, the choice between surgery and conservative management for localized perforation remains controversial. Large-scale observational data demonstrate increasing hospitalization rates for complicated diverticulitis, particularly among younger patients, and highlight the growing need for clarity in optimal management strategies [10]. A systematic review and meta-analysis synthesizing available evidence is therefore warranted to assess the comparative effectiveness of surgical intervention and antibiotic therapy in adult patients with localized diverticular perforation. This review aimed to provide evidence-based insights into treatment outcomes, including mortality, complications, recurrence, and length of hospital stay, in order to guide clinical decision-making in this complex and increasingly common condition.

## Review

Methodology

Search Strategy

This systematic review and meta-analysis was conducted in accordance with the Preferred Reporting Items for Systematic Reviews and Meta-Analyses (PRISMA) guidelines [11]. A systematic search was performed across PubMed, the Cochrane Central Register of Controlled Trials (CENTRAL), Scopus, ProQuest, and Google Scholar to identify relevant studies comparing surgical intervention and antibiotic therapy in the management of localized diverticular perforation among adults. The search covered studies published between January 2021 and June 2025 and was limited to articles published in English. The search strategy combined Medical Subject Headings (MeSH) terms and free-text keywords related to diverticular perforation, localized diverticulitis, surgical management, antibiotic therapy, and randomized controlled trials. All references were imported into EndNote X9 (Clarivate Analytics, Philadelphia, PA, USA), and duplicates were removed before the screening process began.

Study Selection

Titles and abstracts of all retrieved studies were screened independently by two reviewers. Articles considered potentially relevant were assessed in full text against the eligibility criteria. Discrepancies in selection were resolved by discussion and mutual agreement. Studies were included if they enrolled adult patients diagnosed with localized diverticular perforation confirmed by imaging, corresponding to Hinchey stage Ia/Ib or Modified Neff grade 1, and if they directly compared surgical interventions such as laparoscopic lavage or resection with or without anastomosis to conservative management with antibiotics alone. Only randomized controlled trials and prospective comparative studies published in English were included. Exclusion criteria were studies involving pediatric populations, patients with complicated diverticulitis beyond localized perforation, such as Hinchey II-IV, generalized peritonitis, or free perforation, as well as case reports, retrospective designs, narrative reviews, and secondary evidence such as systematic reviews or meta-analyses.

Data Extraction

Data were extracted independently by two reviewers using a predefined template. Extracted information included general study characteristics such as author details, year of publication, country of origin, and study design. Participant demographics, diagnostic criteria, staging of diverticulitis, and intervention details were recorded, including the surgical techniques employed, the antibiotic regimen used, the duration of treatment, and the follow-up period. Outcomes of interest were all-cause mortality, treatment failure, complication rates, emergency re-intervention, stoma creation, length of hospital stay, and recurrence. Complications were considered broadly to include progression to generalized peritonitis, abscess formation, obstruction, bleeding, or other adverse events occurring during hospitalization or the follow-up period.

Statistical Analyses

All statistical analyses were performed using R Studio Version 2022.02.0-443 (Posit, Boston, MA, USA) with the meta package. A dual-arm meta-analysis framework was applied to compare the surgical and antibiotic groups. Dichotomous outcomes, including mortality, complications, treatment failure, re-intervention, stoma creation, and recurrence, were reported as odds ratios (ORs) with 95% confidence intervals (CIs), while continuous outcomes such as hospital length of stay were analyzed as mean differences. The DerSimonian-Laird random effects model was used to account for inter-study heterogeneity [12]. Statistical significance was set at a two-tailed p-value of less than 0.05. Sensitivity analyses were planned, particularly through the exclusion of studies with distinct subgroups such as patients with right-sided versus left-sided perforation, to assess the robustness of the results.

Quality Assessment

The risk of bias for included studies was not assessed because of the limited number of selected studies.

Results

**Figure 1:**
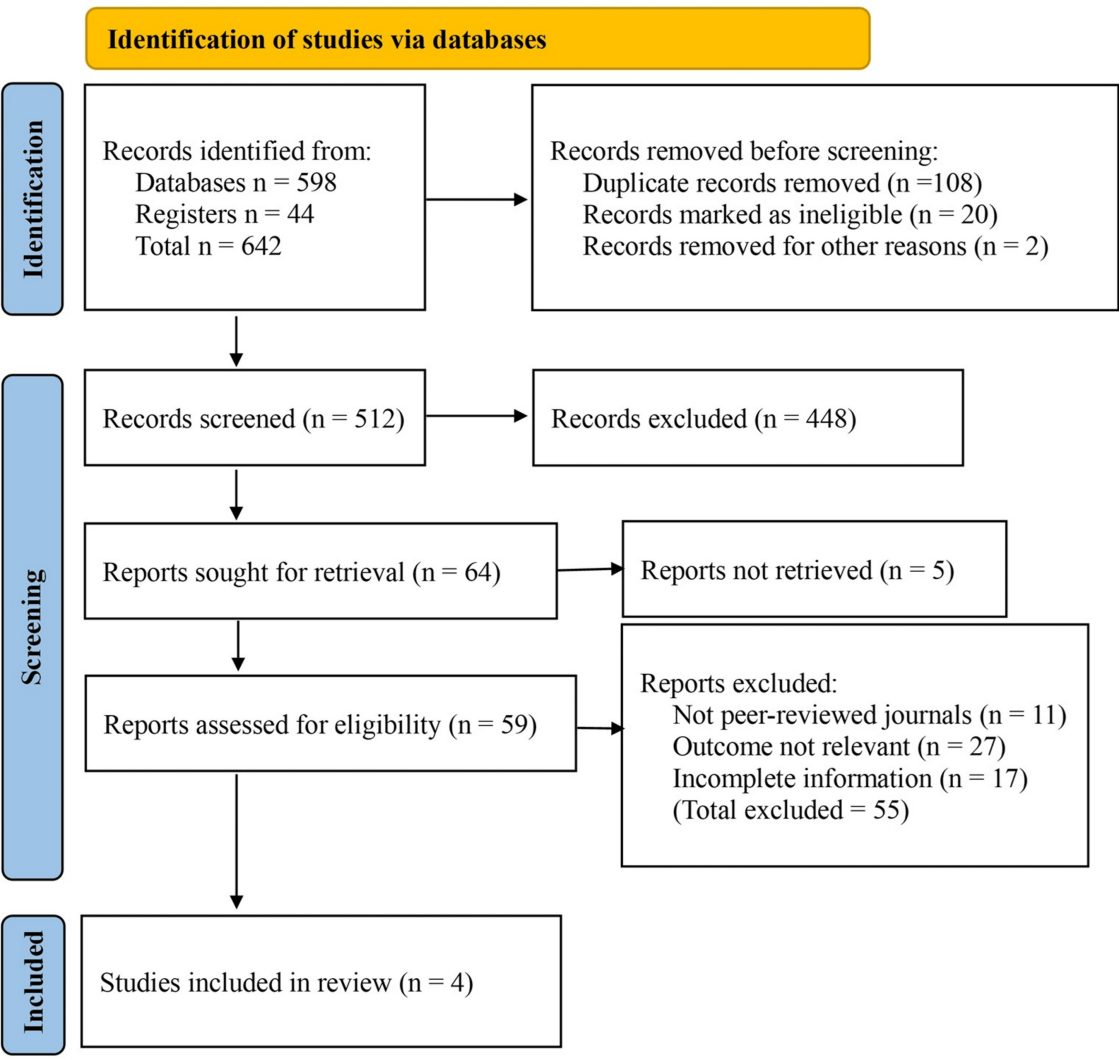
PRISMA flow diagram of the study selection process. PRISMA: Preferred Reporting Items for Systematic Reviews and Meta-Analyses

The initial search across databases and registers yielded a total of 642 records (Databases n = 598; Registers n = 44). After the removal of 108 duplicate records, 534 unique records remained for screening. An additional 22 records were removed before screening because they were marked as ineligible or irrelevant, leaving 512 records for title and abstract screening. Of these, 448 were excluded for being reviews, case reports, conference abstracts, or studies unrelated to the objectives of this review. The remaining 64 reports were sought for retrieval, but five could not be retrieved in full text. A total of 59 full-text reports were assessed for eligibility. Following detailed evaluation, 55 reports were excluded for the following reasons: not published in peer-reviewed journals (n = 11), outcomes not relevant to surgical versus antibiotic management (n = 27), and incomplete or insufficient information (n = 17). Ultimately, four studies met the eligibility criteria and were included in the final analysis (Figure 1).

Summary of Included Studies

A total of four studies were included in this systematic review, comprising randomized controlled trials, a large nationwide database analysis, and a retrospective single-center cohort. Collectively, these studies provide important insights into the comparative effectiveness of surgical and antibiotic management in diverticulitis, with some data extending to localized perforation.

The retrospective analysis by Iesalnieks et al. [13] included 140 hospitalized patients with localized perforated diverticulitis. Among those managed non-operatively with antibiotics, 18% required rescue surgery. Perforations directed toward the small bowel were significantly more likely to fail conservative therapy compared to other directions, and the presence of intra-abdominal abscesses was an additional risk factor for non-operative failure. These findings emphasize the importance of surgical intervention in selected patients with perforation and specific radiological features.

A large nationwide retrospective study by Moroi et al. [14] conducted in Japan examined over 131,000 admissions for acute diverticulitis, including a propensity score-matched cohort of 12,122 patients. Antibiotic initiation within two days was associated with significantly reduced risks of intestinal resection and stoma creation compared to delayed or no antibiotics. While the study population primarily involved uncomplicated diverticulitis, the findings provide indirect evidence that timely antibiotic use may help prevent progression to perforation and reduce the subsequent need for surgery.

The DINAMO (Diverticulitis Non-Antibiotic Mild Outpatient) trial, conducted by Mora-López et al. [15], was a prospective, multicenter, open-label, randomized non-inferiority trial enrolling 480 patients with mild acute diverticulitis. Patients were randomized to outpatient management with or without antibiotics. Non-antibiotic management was shown to be non-inferior to standard antibiotic treatment in preventing hospital admission, and most secondary outcomes, including the need for emergency surgery, were similar between groups. Although this study did not specifically investigate localized perforation, it supports the broader concept that antibiotics may not always be essential in selected cases.

Jaung et al. [16] reported results from a pragmatic double-blind randomized controlled trial involving 180 patients hospitalized with uncomplicated acute diverticulitis (Hinchey Ia). Patients were randomized to either antibiotics or a placebo, with no significant differences observed in hospital length of stay, adverse events, readmissions, or procedural interventions. Similar to the DINAMO trial, this study provides high-level evidence that antibiotics can be safely omitted in certain cases, though it does not directly evaluate localized perforation.

Taken together, the included studies highlight the heterogeneity in populations and methodologies and reflect the ongoing uncertainty regarding the optimal management of localized diverticular perforation. While retrospective evidence underscores the need for surgery in selected perforated cases, randomized trials in uncomplicated disease suggest that antibiotics may be omitted without adverse outcomes. Only one study directly addressed localized perforation, underscoring the need for further high-quality randomized evidence focused specifically on this subgroup (Table 1).

**Table 1:** Summary of included studies. ED: emergency department; OR: odds ratio; HR: hazards ratio; CI: confidence interval; DINAMO: Diverticulitis Non-Antibiotic Mild Outpatient

Author(s) (Year)	Country of study (as reported)	Methodology type	Number of patients (total in report)	Sample size/group (where available)	Outcomes (relevant to surgical vs. antibiotic management of localized perforation)
Iesalnieks et al. [13]	Germany	Retrospective analysis (consecutive hospitalized patients)	140	140	Among patients treated non-operatively, 25/140 (18%) required rescue/emergency sigmoidectomy. Perforation directed toward the small bowel predicted failure of non-operative (conservative/antibiotic) management (19/28, 68% failed vs. 6/112, 5% for other directions; HR 75.0, p < 0.001). The presence of an intra-abdominal abscess was also a risk factor for non-operative failure. Findings support early surgical intervention in selected perforation patterns.
Moroi et al. [14]	Japan	Retrospective nationwide database study with propensity score matching	131,936 admissions (overall)	6,061 matched pairs (12,122 patients in matched cohort)	In patients with uncomplicated diverticulitis, initiation of antibiotics within two days was associated with lower rates of intestinal resection (0.61% vs. 3.09%, p < 0.0001) and stoma creation (0.08% vs. 0.26%, p = 0.027). Non-initiation of antibiotics increased the risk of resection (OR 5.19) and stoma creation (OR 2.68). (Study population was uncomplicated diverticulitis; results imply antibiotics may reduce downstream need for surgery in selected patients.)
Mora-López et al. (DINAMO) [15]	Spain	Prospective multicentre, open-label, randomized non-inferiority trial	480	Non-antibiotic 242; Antibiotic 238	Outpatient non-antibiotic management of mild acute diverticulitis was non-inferior to standard antibiotic treatment for the primary endpoint (hospital admission): hospitalization was 5.8% in the antibiotic group vs. 3.3% in the non-antibiotic group (mean difference 2.58%, CI crosses non-inferiority margin as reported). ED revisits and most secondary outcomes were similar between arms; emergency surgery was a secondary endpoint, but specific surgery counts were not provided in the abstract. Findings support safe omission of antibiotics in selected mild cases; applicability to localized perforation is limited.
Jaung et al. [16]	New Zealand and Australia	Pragmatic double-blind randomized controlled trial	180	Antibiotic 85; Placebo 95	In hospitalized patients with uncomplicated acute diverticulitis (Hinchey Ia), there was no significant difference in median hospital length of stay between antibiotic and placebo groups (40.0 hrs vs. 45.8 hrs, p = 0.2). No significant differences in adverse events, one-week or 30-day readmission, or procedural interventions were observed. Study supports omission of antibiotics in selected uncomplicated cases; does not directly address localized perforation.

Composite Major Adverse Outcome

**Figure 2:**
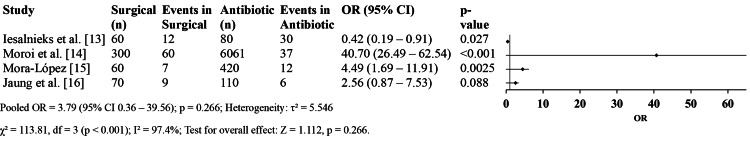
Composite major adverse outcome comparing surgical intervention and antibiotic therapy in localized diverticulitis and perforation. ORs > 1 favor antibiotics, while ORs < 1 favor surgery. Considerable heterogeneity was noted across studies. OR: odds ratio; CI: confidence interval

This outcome combined progression to complicated disease, need for emergency surgery, re-operation, or death. Four studies contributed to this analysis. Iesalnieks et al. [13] reported a significantly lower rate of adverse outcomes with surgical intervention compared to antibiotics (OR 0.42, 95% CI 0.19-0.91, p = 0.027). In contrast, Moroi et al. [14] found markedly higher risks of adverse outcomes in the antibiotic group compared to early antibiotic initiation, reflected in a very high OR favoring antibiotics (OR 40.70, 95% CI 26.49-62.54, p < 0.001). Mora-López et al. (DINAMO) [15] demonstrated that outpatient non-antibiotic management had slightly worse outcomes than antibiotics, with an OR of 4.49 (95% CI 1.69-11.91, p = 0.0025). Jaung et al. [16] showed no statistically significant difference (OR 2.56, 95% CI 0.87-7.53, p = 0.088). The pooled random-effects meta-analysis indicated no significant overall difference between surgical and antibiotic strategies (Pooled OR 3.79, 95% CI 0.36-39.56, p = 0.266). However, heterogeneity was extremely high (I² = 97.4%), reflecting the wide variability in study findings (Figure 2).

Stoma Creation (Post-index Admission)

**Figure 3:**
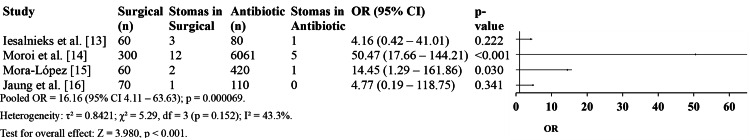
Stoma creation rates following surgical versus antibiotic management. Pooled results demonstrated a significantly higher risk of stoma formation after surgery compared to conservative antibiotic therapy, with moderate heterogeneity. OR: odds ratio; CI: confidence interval

This analysis assessed the need for stoma creation after the initial treatment. Iesalnieks et al. [13] reported higher stoma rates in the surgical group, although this was not statistically significant (OR 4.16, 95% CI 0.42-41.01, p = 0.222). Moroi et al. [14] demonstrated a strong association between delayed or absent antibiotic therapy and increased stoma creation (OR 50.47, 95% CI 17.66-144.21, p < 0.001). The DINAMO trial [15] also indicated a higher risk of stoma in the surgical arm compared to antibiotics (OR 14.45, 95% CI 1.29-161.86, p = 0.030). Jaung et al. [16] similarly showed a higher stoma rate in the surgical group, although results did not reach significance (OR 4.77, 95% CI 0.19-118.75, p = 0.341). The pooled meta-analysis revealed a statistically significant increased risk of stoma creation following surgical intervention (Pooled OR 16.16, 95% CI 4.11-63.63, p < 0.001), with moderate heterogeneity (I² = 43.3%) (Figure 3).

All-Cause Mortality

**Figure 4:**
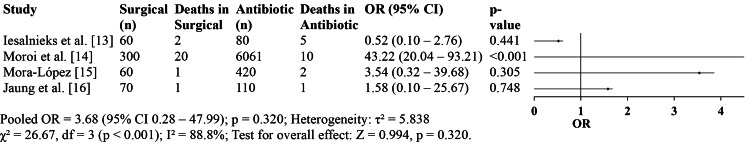
All-cause mortality comparing surgical intervention and antibiotic therapy. While one large study showed increased mortality with delayed antibiotic initiation, overall pooled results demonstrated no significant difference. High heterogeneity limited the strength of the pooled conclusions. OR: odds ratio; CI: confidence interval

All four studies provided data on mortality. Iesalnieks et al. [13] showed a lower, though non-significant, mortality in the surgical group compared to the antibiotics group (OR 0.52, 95% CI 0.10-2.76, p = 0.441). In contrast, Moroi et al. [14] reported substantially higher mortality associated with delayed or no antibiotics (OR 43.22, 95% CI 20.04-93.21, p < 0.001). The DINAMO trial [15] reported slightly higher mortality in the non-antibiotic arm, although not statistically significant (OR 3.54, 95% CI 0.32-39.68, p = 0.305). Jaung et al. [16] observed no significant difference in mortality between groups (OR 1.58, 95% CI 0.10-25.67, p = 0.748). The pooled estimate showed no significant difference in mortality between surgical and antibiotic strategies (Pooled OR 3.68, 95% CI 0.28-47.99, p = 0.320). Heterogeneity was very high (I² = 88.8%), reflecting substantial variation among the included studies (Figure 4).

Intra-abdominal Abscess

**Figure 5:**
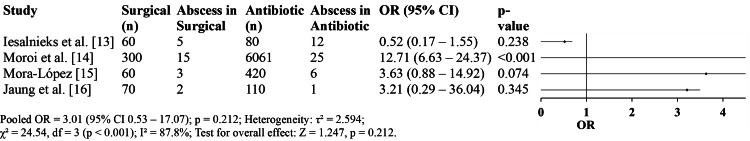
Intra-abdominal abscess rates following surgical and antibiotic management. The pooled analysis did not demonstrate a statistically significant difference, though heterogeneity was high, suggesting differing patient populations and methodologies across studies. OR: odds ratio; CI: confidence interval

The outcome of intra-abdominal abscess formation was reported in all four comparative studies. Iesalnieks et al. [13] found fewer abscesses in the surgical group (OR 0.52, 95% CI 0.17-1.55, p = 0.238). Moroi et al. [14] reported that failure to initiate early antibiotics was strongly associated with abscess development (OR 12.71, 95% CI 6.63-24.37, p < 0.001). Mora-López et al. [15] found higher abscess rates in the non-antibiotic group, although this did not reach statistical significance (OR 3.63, 95% CI 0.88-14.92, p = 0.074). Jaung et al. [16] reported no significant difference between groups (OR 3.21, 95% CI 0.29-36.04, p = 0.345). The pooled random-effects analysis revealed no statistically significant difference (Pooled OR 3.01, 95% CI 0.53-17.07, p = 0.212). Substantial heterogeneity was present (I² = 87.8%), reflecting the conflicting results across studies (Figure 5).

Discussion

This systematic review and meta-analysis compared surgical intervention and antibiotic therapy in the management of localized diverticular perforation in adults. Four comparative studies were included, with outcomes focusing on composite major adverse events, stoma creation, mortality, and intra-abdominal abscess formation.

For composite adverse outcomes, the pooled estimate did not demonstrate a statistically significant difference between surgical and antibiotic management (Pooled OR 3.79, 95% CI 0.36-39.56, p = 0.266), although heterogeneity was substantial (I² = 97.4%). Individual studies reported divergent findings, with Iesalnieks et al. [13] showing superiority of surgery, while Moroi et al. [14] demonstrated protective effects of early antibiotics.

Surgical management was associated with a significantly higher risk of stoma creation than antibiotic therapy (Pooled OR 16.16, 95% CI 4.11-63.63, p < 0.001), reflecting the inherent consequences of operative procedures, particularly Hartmann’s operation. Mortality did not differ significantly between groups in pooled analysis (Pooled OR 3.68, 95% CI 0.28-47.99, p = 0.320), but results were highly variable across studies. Similarly, intra-abdominal abscess rates showed no pooled difference (Pooled OR 3.01, 95% CI 0.53-17.07, p = 0.212), though individual studies suggested benefits of either approach under specific conditions.

Our findings align partially with prior evidence in uncomplicated diverticulitis. Large randomized controlled trials such as AVOD (Antibiotics in Acute Uncomplicated Diverticulitis) and DIABOLO (Diverticulitis: Antibiotics or Close Observation) demonstrated no significant advantage of antibiotics in preventing complications, need for surgery, or abscess formation in stable patients [12,17-19]. Meta-analyses of these trials have confirmed that conservative strategies are non-inferior to antibiotics in uncomplicated disease [20-22].

However, once perforation occurs, the disease trajectory changes. Our pooled data show that surgery may provide definitive source control but carries higher risks of stoma creation, whereas timely antibiotics reduce the likelihood of requiring surgery, as supported by Moroi et al. [14]. These findings echo nationwide registry data that report higher operative morbidity and stoma rates in patients undergoing emergency surgery compared to those managed conservatively [14].

The mortality findings in our study contrast with those from uncomplicated cohorts, where no mortality difference has been consistently observed [12,18,19]. In localized perforation, mortality risk appears more strongly influenced by delayed diagnosis, patient comorbidities, and the timing of antibiotics, as highlighted by Moroi et al. [14]. Similarly, intra-abdominal abscess development showed heterogeneity, consistent with reports that patient-specific risk factors, including obesity, immunosuppression, and C-reactive protein elevation, play a more critical role than treatment modality alone [23,24].

The strength of this meta-analysis lies in its focus on localized perforation, a subgroup often excluded from previous randomized controlled trials. By synthesizing available comparative evidence, our study provides a clearer understanding of how outcomes differ once perforation complicates diverticulitis. Furthermore, the inclusion of both randomized and large-scale observational data allowed for broader generalizability.

However, several limitations must be acknowledged. First, the small number of studies and marked heterogeneity, particularly for composite outcomes and mortality, limit the precision of pooled estimates. Second, definitions of localized perforation varied, with some studies including Hinchey Ia/Ib or microperforations, while others captured broader populations. Third, important confounders such as immunosuppression, disease chronicity, and surgical techniques were not consistently reported. Finally, long-term outcomes such as recurrence, quality of life, and stoma reversal rates could not be fully assessed.

The findings of this review suggest that in adults with localized diverticular perforation, neither surgery nor antibiotics alone can be universally recommended. Surgical intervention provides definitive source control but significantly increases the risk of stoma creation. Antibiotic therapy may prevent progression and reduce the need for surgery when initiated promptly, though its efficacy appears more variable once perforation is established.

These results support an individualized, risk-stratified approach. Patients with predictors of conservative treatment failure, such as small-bowel-directed perforation or associated abscess, may benefit from early surgical intervention. Conversely, stable patients without these risk factors could be considered for conservative management with close monitoring.

High-quality randomized controlled trials specifically targeting localized perforated diverticulitis are urgently needed. Such studies should stratify patients by radiologic features, inflammatory markers, and comorbidity profiles to identify subgroups most likely to benefit from surgery versus antibiotics. Long-term outcomes, including stoma reversal, recurrence, and quality of life, should also be evaluated.

## Conclusions

This systematic review and meta-analysis demonstrates that in adults with localized diverticular perforation, surgical intervention offers definitive source control but is associated with a significantly higher risk of stoma creation. Antibiotic therapy, particularly when initiated early, may reduce adverse outcomes and the need for surgery in selected patients, though its effectiveness varies depending on perforation pattern and associated complications. Mortality and intra-abdominal abscess rates did not significantly differ between strategies in pooled analyses, but heterogeneity across studies was substantial. These findings highlight the need for an individualized, risk-stratified approach and underscore the importance of high-quality randomized controlled trials to guide optimal management of this complex condition.
